# A quantitative assessment of current practice in diabetes and hypertension services in pharmacies in urban Nepal

**DOI:** 10.1371/journal.pone.0328827

**Published:** 2025-07-18

**Authors:** Grishu Shrestha, Deepak Joshi, Parash Mani Sapkota, Sampurna Kakchapati, Bryony Dawkins, Helen Elsey, Shreeman Sharma, Abhigyna Bhattarai, Sushil Chandra Baral

**Affiliations:** 1 HERD International, Lalitpur, Nepal; 2 Academic Unit of Health Economics, Leeds Institute of Health Sciences, University of Leeds, Leeds, United Kingdom; 3 Hull and York Medical School, University of York, United Kingdom; Patan Academy of Health Sciences, NEPAL

## Abstract

**Background:**

Community pharmacies are one of the first contact points for essential health care in Nepal particularly for those living in cities with limited access to primary care. With increasing prevalence of non-communicable diseases there is increasing recognition that pharmacies should be competent and well-equipped to provide basic services for clients with common NCDs. This paper aims to assess the hypertension and diabetes services delivered by pharmacies and their readiness to deliver quality services in Pokhara Metropolitan City (PMC).

**Methods:**

We identified all pharmacies providing hypertension and diabetes services in the 33 wards of PMC and gained their consent to complete an adapted version of World Health Organization’s Service Availability and Readiness Assessment (SARA) and National Health Facility Survey 2015. The adapted tool assessed the characteristics, extent and quality of diabetes and hypertension services provided within the pharmacies. Readiness to deliver hypertension services was assessed across two domains: i) trained human resource and guidelines and ii) available drugs and supplies; with the addition domain of iii) diagnostics for diabetes. Data analysis was conducted using R studio (version 4.3.0).

**Results:**

A total of 352 private pharmacies were identified in PMC, 94% of which were registered. The main provider of services was a pharmacist with a paramedic background (37%). While all pharmacies were providing provisional, definitive diagnosis and treatment for diabetes and hypertension, few were categorized as ‘ready’ to deliver appropriate and quality diabetes or hypertension services, with a mean readiness score of 30.7 for diabetes and 32.1 for hypertension. The average score was highest for the essential equipment and supplies domain for diabetes whereas for hypertension services it was comparatively lower. There were no statistically significant differences in service readiness for both diabetes and hypertension between pharmacies with and without paramedics.

**Conclusion:**

In urban areas, private pharmacies are involved in the management of non-communicable diseases particularly diabetes and hypertension, but they are poorly resourced, with little guidance and training. Given the continued lack of sufficient public primary care services to meet the needs of the growing urban population, comprehensive training to enhance the capacity of pharmacy staff in the management of diabetes and hypertension, aligning with WHO’s Package of Essential NCD (PEN) guidelines is needed. Standardized clinical protocols, strengthened regulatory oversight through registration, licensing and continuous monitoring will further support quality service provision.

## Background

Universal Health Coverage (UHC) articulates the global goal that all should have access to quality health services without suffering financial hardship. This includes the full range of services including health promotion, prevention, treatment, rehabilitation and palliative care [1–3]. UHC is a key component of Sustainable Development Goal 3, (specifically target 3.8) which focuses on health [4]. These global goals aim to increase access to healthcare for all individuals and set minimum standards, through effective training, guidelines, rules, regulations, and financing [1]. However, rapid urbanization and changing disease burdens has placed great pressure on primary care systems in low income countries. Increasingly there are calls to establish partnerships with non-state providers [5] as without this collaboration, the goals of UHC will remain illusive, with urban poor communities facing inequity in access and outcomes [6].

This pressure on primary care comes not only from the increasing numbers of urban residents, but also from the changing disease burden. The nutrition transition along with changes in levels of physical activity found in urban areas have combined to dramatically increase the prevalence of Non-communicable Diseases (NCDs) [7,8]. NCDs account for about 41 million deaths worldwide, equivalent to 74% of all fatalities [9]. The four main NCDs - cardiovascular diseases (CVDs), diabetes mellitus, cancers and respiratory diseases – account for more than 86% of all premature deaths linked to NCDs in LMICs [9,10]. Similar to other LMICs, Nepal is also dealing with the high burden of NCDs, with 71% of all deaths in 2019 due to NCDs [11]. According to the WHO STEPwise approach to surveillance (STEPS) survey 2019, there was a 24.5% prevalence of hypertension and 5.8% prevalence of diabetes in Nepal [12]. Diabetes and cardiovascular disease are responsible for the highest number of NCD deaths, whilst hypertension is one of the most common CVDs, with its prevalence rising significantly across LMICs [13,14].

While urban health policy in Nepal has pays attention to preventive measures to address NCDs [15] and the Government of Nepal has developed national protocols based on WHO’s PEN package to manage NCDs, there is still a significant implementation gap. For example, a qualitative assessment found limited attention paid to NCDs risk factors with services focused on curative rather than preventative interventions as well as challenges with overburdened and unskilled health works, challenges with diagnostic equipment and NCDs medications and an absence of NCDs monitoring and reporting [16]. Quantitative assessments have also found a lack of preparedness in both rural and urban areas, with only 6% of facilities in Syangja district being identified as prepared to respond to the burden of NCDs [17].

The antagonism of these two factors: a growing urban population and increasing burden of NCDs has left the urban, public primary health system overstretched and unable to meet population needs. Low-income urban residents who rely on the free or low-cost services of the public sector have turned instead to a plethora of private providers. In particular, small, private pharmacists have sprung up to meet demand [18,19].

Although private pharmacies form part of a UHC system, a systematic review of the performance of pharmacies in south Asia found multiple challenges relating to quality, including: i) poor history taking practice, ii) failure to refer patients requiring medical attention, iii) illegal sale of prescription-only medicines without prescriptions, iv) irrational use of drugs and v) inadequate information or counseling provided to patients [20]. Despite this inconsistent and often poor quality, pharmacies are one of the first contact point for urban communities due to opening hours that fit with the long working days of low-income urban residents and their knowledge and friendliness towards the communities in which they reside [5]. Increasingly it is recognized that pharmacies have an important role in prevention and management of NCDs through offering direct interventions such as medication education and management of disease resulting in improvement of medication adherence and promotion of safe medication practices [21]. Pharmacists in many low, middle and high-income countries are now involved in many areas of healthcare delivery such as ensuring the safe and quality use of medicines, promoting public health and providing primary healthcare services [22].

Community pharmacies are one of the key private providers of essential health care across Nepal and are one of the first contact points for people in the community. They provide services such as medicines dispensing along with the relevant health information and check-ups [23]. Today there are a total of 29,948 pharmacies registered at Department of Drug Administration (DDA) in Nepal [24]. According to a national private sector survey of child health services, community pharmacies are run by paramedical health professionals (75%) with diploma certification including health assistants, nurses, besides the pharmacists [25]. In 2005 the Nepal Government drafted the National Good Pharmacy Practice Guidelines to refine the practice of pharmacies. This has expanded the provision of services delivered by pharmacies to include counselling patients, first aid services, monitoring blood pressure, medicines record keeping and vaccination [24,26]. Given the growth in the pharmacy sector, particularly the increasing number of assistant pharmacists and the reliance of the population on self-medication it is expected that guidelines will be updated in the near future [26]. Currently, there are 42 colleges who provide Diploma in Pharmacy (D. Pharm) and 27 colleges providing Bachelor’s in Pharmacy (B. Pharma) education which produce more than 2,500 graduates annually in Nepal [26–28]. Even if they are employed but their professional skills are not being used [26].

Given the gaps in public primary care in urban areas and the trust clients already place in them, pharmacies have the potential to play a valuable role in providing accessible NCD services. Within the current legislation in Nepal, this could include screening, advice and referral when needed. Understanding current with quality improvements, to meet the growing healthcare needs of the population, pharmacies should be competent and well-equipped to provide basic health services, including NCDs services. Though pharmacies provide some level of care for NCDs patients, there is lack of clear evidence about the extent and nature of the services they provide. Thus, this study aims to assess the hypertension and diabetes services delivered by pharmacies and their readiness to deliver quality services as defined by the WHO Service Availability and Readiness Assessment (SARA) criteria.

## Methods

### Study design

The study deployed a cross-sectional census survey conducted in all pharmacies of PMC. The study followed STROBE guidelines for observational studies.

### Study setting

Pokhara Metropolitan City (PMC) in Nepal’s Gandaki Province is the largest metropolitan city of the country by area, covering a total land area of 464.24 km^2^, and is located in the western part of the country. According to the Central Bureau of Statistics (CBS) 2021, there are 518, 452 people residing in the 33 wards of PMC, which has increased from 324,489 in 2011 with the growth rate of 59.7% [29,30].

### Participants

#### Eligibility criteria.

All the private community pharmacies of PMC were included and any pharmacy within either a public or private hospital was excluded.

### Outcome variables and measurement

Questions from the WHO SARA manual which provided data on services available and the readiness of the pharmacy to provide the NCDs related services were selected and included in the survey administered to pharmacies across PMC.

### Data collection

The census method was used to collect data from pharmacies throughout the 33 wards of the PMC. The data collection was done from 29^th^ March 2022–24^th^ April 2022. Altogether 20 field researchers (FR) were recruited who were graduate in health sciences. Total 10 teams were formed, with two FRs in each team and they were allotted 3–4 wards. In each ward they held discussion with key informants to list the pharmacies, and in each pharmacy the team visited, they obtained written consent for the interview. Following the completion of the interview, they further validated the list at each pharmacy to ensure they are not missing any pharmacies in the list. Researchers (DJ, RRN and SG) also validated the list with open street map. Two sets of tools were developed; one including the basic information including their infrastructure, registration, graphic information system (GIS) data, types of services provided. If the pharmacies were providing hypertension or diabetes related services beyond drug dispensing, then second set of tools was administered to further assess the readiness and availability of the services following the WHO SARA tool [31]. Quantitative data were collected based on the health facility assessment tool which was adapted from the standard WHO questionnaire [31] and Nepal Health Facility Survey (NHFS) 2015 [32]. The tools capturing the information on service availability and readiness were adapted from Nepal Health Facility Survey (NHFS) 2015 that had been developed by the DHS program in consultation with national level stakeholders. In addition, we performed tool pretesting in 10 pharmacies and held one round of stakeholder meeting to finalize the tools before implementing in the field.

### Outcome measurement

#### Service readiness.

A customized version of SARA model, developed by the WHO, was used in assessing the service readiness of the pharmacies [33]. Domains and items that are relevant for screening and for primary diagnosis and counseling/health message delivery that are relevant for pharmacies were utilized to assess service readiness. Specific indicators measured the responsiveness of pharmacies to offer the relevant services across three domains: i) existing guidelines and training provided, ii) presence of essential equipment and supplies, and iii) performing diagnostic tests for diabetes mellitus which is mentioned in Tables 1 and 2. This approach is consistent with the WHO PEN package which emphasizes the importance of readying primary healthcare facilities in rendering basic services for the prevention and control of noncommunicable diseases such as hypertension and diabetes.

**Table 1:** Measurement of Hypertension service readiness.

Staff and Guidelines	T1	Availability of Human Resource trained (within the last two years) in management of CVDs	No = 0 Yes = 1	Staff and Guidelines Readiness Score = $\frac{T1+T2}{2}$
	T2	Availability of management guidelines	No = 0 Yes = 1	
Equipment	T3	Availability of functioning BP set	No = 0 Yes = 1	Equipment Readiness Score = $\frac{T3+T4+T5+T6+T7}{5}$
	T4	Availability of weight measuring equipment	No = 0 Yes = 1	
	T5	Availability of CVD risk chart	No = 0 Yes = 1	
	T6	Availability of IEC materials for hypertension	No = 0 Yes = 1	
	T7	Availability of stethoscope	No = 0 Yes = 1	
Hypertension Service Readiness = $\frac{Staff\;and\;Guideline\;Readiness\;Score\;+\;Equipment\;Readiness\;Score}{2}$				

**Table 2:** Measurement of Diabetes service readiness.

Staff and Guidelines	T1	Availability of Human Resource trained (within the last two years) in management of Diabetes	No = 0 Yes = 1	Staff and Guidelines Readiness Score = $\frac{T1+T2}{2}$
T2	Availability of management guidelines	No = 0 Yes = 1
Equipment	T3	Availability of functioning BP set	No = 0 Yes = 1	Equipment Readiness Score = $\frac{T3+T4+T5+T6+T7}{5}$
	T4	Availability of weight measuring equipment	No = 0 Yes = 1	
	T5	Availability of height measuring equipment	No = 0 Yes = 1	
	T6	Availability of IEC materials for diabetes	No = 0 Yes = 1	
	T7	Availability of stethoscope	No = 0 Yes = 1	
Diagnostic Services	T8	Blood Glucose	No = 0 Yes = 1	Diagnostic Services Readiness Score = $\frac{T8+T9+T10+T11}{4}$
	T9	Urine Protein	No = 0 Yes = 1	
	T10	Urine Ketone	No = 0 Yes = 1	
	T11	Lipid Profile	No = 0 Yes = 1	
Diabetes Service Readiness = $\frac{Staff\;and\;Guideline\;Readiness+\;Equipment\;Readiness+\;Diagnostic\;Services\;Readiness}{3}$				

The readiness score was calculated based on the availability of items. The items in each domain were recoded as binary variables, with ‘1’ denoting the item’s presence and ‘0’ denoting its absence in the facility. The sum of the scores for each item was divided by the total number of items, and the result was multiplied by 100 to determine the mean score for each domain. The overall preparedness score is influenced equally by each of the scoring domains. The preparedness score was the average score across the three domains [34]. Readiness to deliver diabetes services and hypertension management services was assessed separately in each pharmacy included in the study.

### Pharmacy characteristics

The following data were collected for each pharmacy surveyed whose operational definition has been provided in Table 3: the setting (urban/ peri urban/rural), providers of care based on the HR (human resource) roster (non- paramedic/ paramedic/ pharmacist with paramedic background/ pharmacist without paramedic background), whether pharmacies carried out quality assurance activities routinely (yes = 1/no = 0), the registration status of the pharmacies, whether a system to record the health service data was available (yes = 1/ no = 0), whether the pharmacy is linked with PMC (yes = 1/ no = 0), and, whether pharmacies provide hypertension services and diabetes services (yes = 1/no = 0).

**Table 3:** Operational Definitions.

Urban and Peri- urban/rural area	Urban and peri-urban/rural wards of PMC were categorized based on the Degree of Urbanization in Nepal Report [35].
Paramedics	A paramedic is a trained medical professional who acquires diploma certification from accredited institute. Paramedics can examine, evaluate and treat patients with equipment and medications. In Nepal, staff nurses, health assistants, auxiliary health workers, auxiliary nurse midwives, and are usually called paramedics [36,37].
Pharmacist	Pharmacists are those individuals who hold bachelor’s degree or diploma in pharmacy [38].
Paramedic pharmacist	Pharmacists are those personnel who hold a diploma or bachelor’s degree in pharmacy [38]. Pharmacists who also have a paramedic background are included in this category.
Non- paramedics	The DDA has deemed persons who had taken a 45- hour course or training, as capable for running a pharmacy [39], for this study we have termed this category ‘non-paramedics’. This category also includes pharmacy staff without either paramedic, paramedic-pharmacists or pharmacist training.
Quality assurance	Quality assurance is a technique used to ensure quality of practice and its outcome in pharmacy.
Registration	Those pharmacies who have registered their pharmacies in either one of the organizations, i.e., Ministry of Health and Population, Department of Health Service, Department of Drug Administration, and PMC is considered as registered. It is mandatory for the pharmacy to register in either of these organizations to operate the pharmacy, and they need to renew this annually The pharmacy can only be registered under the name of pharmacist and pharmacy assistant along with responsibility of operating it by either of the personnel [37].
Recording of health data	Recording of health data includes systematic method of collecting health service data within the pharmacy
Linkage with PMC	Linkages with PMC could be in the form of participation in the training or capacity building activities, data reporting, participation in review meeting.
Provisional diagnosis	Provisional diagnosis is the first considered diagnosis which initiates the first phase of management [40].
Definitive diagnosis	A final diagnosis that is made after getting the results of tests, such as blood tests and biopsies, that are done to find out if a certain disease or condition is present [41].

### Analysis

Descriptive Statistics were used to draw out insights about current service provision and service readiness according to pharmacy characteristics outlined above. Data were extracted from the Open Data Kit (ODK) tool into the R studio (version 4.3.0) to be cleaned and analyzed. We used base R functions for computations, *dplyr* package for data transformation and *gtsummary* for data tabulation.

Mean scores for the domains of guidelines and training, essential equipment and supplies, and diagnostic services were computed by dividing the total score within each domain by the number of corresponding tracer items, as outlined in Table 1 and Table 2. Standard deviations (SD) were also calculated. To obtain the overall mean service readiness score, the cumulative scores from each domain were averaged by dividing by the total number of assessed domains.

Welch Two Sample t-test was conducted to compare the mean readiness scores for diabetes and hypertension services across peri-urban/rural and urban pharmacies and pharmacies with or without paramedic personnel.

### Ethics statement

The ethical approval was taken from Nepal Health Research Council under the reference number 1435 and from University of Leeds under the reference number MREC 21−016.

## Results

### Characteristics of private pharmacies in PMC

A total of 352 private pharmacies were identified in PMC (Table 4). All eligible pharmacies were included in the study and completed the survey. Almost three quarters (75%) of all pharmacies in PMC were in the urban areas with the rest in peri-urban areas.

**Table 4:** Characteristics of private pharmacies in PMC.

Characteristics	Number (N = 352)	Percentage (%)
**Residence**		
Peri urban/ Rural	89	25.28
Urban	263	74.72
**Human Resource**		
Non-paramedic	18	5.11
Paramedic	98	27.84
Pharmacist	107	30.40
Paramedic Pharmacist	129	36.65
**Routine Quality Assurance Activities**		
No	119	33.81
Yes	233	66.19
**Registration Status**		
No	20	5.68
Yes	332	94.32
**System to record Health Service Data**		
No	314	89.2
Yes	38	10.8
**Linkage with PMC**		
No	311	88.35
Yes	41	11.65
**Hypertension Services**		
No	47	13.35
Yes	305	86.65
**Diabetes Services**		
No	179	50.85
Yes	173	49.15

Pharmacists with a paramedic background were the largest group of care providers (37%), followed by paramedic background (28%). Non-paramedics were a smaller proportion of care providers, comprising pharmacist (30%) and non-paramedics (5%). More than one third (34%) of the pharmacies did not conduct any routine quality assurance activities such as checking the sell-by date of medications etc. Whereas the majority (94%) of the pharmacies were registered. Out of the total pharmacies only 11% have a system for recording health related data. Only 12% have a linkage with PMC such as participation in the training or capacity building. The majority of pharmacies (87%) provided hypertension services whilst almost half (49%) provided diabetes services.

### Diabetes services provided by pharmacies

A summary of diabetes services provided by pharmacies is presented in Table 5. Out of the total pharmacies providing diabetes services (n = 173), 15% did not provide any diagnostic services, while the majority of pharmacies (67%) provided provisional diagnostic services only. Of those that provided definitive diagnosis (n = 31, 11%), the majority also provided treatment.

**Table 5:** Diabetes services provided by pharmacies.

Services provided	Number (N = 173)	Percentage (%)
Provisional diagnosis	116	67.05
Definitive diagnosis	12	6.94
Definitive diagnosis and treatment	19	10.98
No diagnosis services	26	15.03

### Hypertension services provided by pharmacies

A summary of hypertension services provided by pharmacies is presented in Table 6. Out of the total pharmacies providing hypertension services (n = 305), 14% did not provide any diagnostic services, while the majority of pharmacies (76%) provided provisional diagnostic services only. Of those that provided definitive diagnosis (n = 28, 3%), the minority provided treatment service as well.

**Table 6:** Hypertension services provided by pharmacies.

Characteristics	Number (N = 305)	Percentage (%)
Provisional diagnosis	233	76.39
Definitive diagnosis	20	6.56
Definitive diagnosis and treatment	8	2.62
No diagnosis services	44	14.43

### Private pharmacy readiness to provide diabetes and hypertension services

Tables 7 and 8 show the distribution of private pharmacy readiness to provide diabetes and hypertension services. Out of the 173 private pharmacies that provided diabetes services, the average readiness score was 30.7 which was slightly lower than the average readiness score for hypertension services (32.1) among 305 private pharmacies. The domain with the least scores for both diabetes and hypertension was the guidelines and trained staff domain with an average score of 1.2 in diabetes services and 0.7 in hypertension services. In contrast to this the average score for the essential equipment and supplies domain was higher for diabetes (70.1) whereas for the hypertension services it was lower (63.6). However, the diagnosis domain was lower for the diabetes services with 21.0 score.

**Table 7:** Readiness of private pharmacies to provide diabetes services.

Characteristics	n%	Score*
**Guidelines and training**		
Trained staff	4(2.3)	1.2(7.5)
Guidelines	0(0.0)	
**Essential equipment and supplies**		
BP apparatus	171(98.8)	70.1(15.5)
Weighing scale	163(94.2)	
Height board	51(29.5)	
IEC materials	50(28.9)	
Stethoscope	171(98.8)	
**Diagnosis**		
Blood Glucose	104(60.1)	21.0(25.3)
Urine Protein	15(8.7)	
Urine Ketone	12(6.9)	
Lipid Profile	14(8.1)	
Overall readiness score*	30.7(11.0)	

* Mean(SD)

**Table 8:** Readiness of private pharmacies to provide hypertension services.

Characteristics	n%	Score*
**Guidelines and training**		
Trained staff	4(1.3)	0.7(5.7)
Guidelines	0(0.0)	
**Essential equipment and supplies**		
BP apparatus	301(98.7)	63.6(12.5)
Weighing scale	283(92.8)	
CVD risk chart	1(0.3)	
IEC materials	84(27.5)	
Stethoscope	301(98.7)	
Overall readiness score*	32.1(7.1)	

* Mean(SD)

### Comparison of diabetes and hypertension service readiness among pharmacies with and without paramedics

Tables 9 and 10 show the comparison of diabetes and hypertension service readiness between pharmacies with and without paramedics. Although pharmacies with paramedics showed slightly higher mean scores particularly in the diagnosis domain for diabetes but these differences were not statistically significant and there were no statistically significant differences in service readiness for both diabetes and hypertension between pharmacies with and without paramedics.

**Table 9:** Comparison of diabetes service readiness scores in pharmacies with and without paramedics.

Characteristic	Pharmacy with paramedics, (N = 110)	Pharmacy without paramedics, (N = 63)	t-statistic	p-value^*1*^
**Diabetes Service Readiness**				
Guidelines and training, Mean (SD)	1.4 (8.2)	0.8 (6.3)	0.51	0.61
Essential equipment and supplies, Mean (SD)	69.1 (14.2)	71.7 (17.5)	−1.03	0.31
Diagnosis, Mean (SD)	23.4 (28.2)	16.7 (18.5)	1.89	0.060
Overall readiness score, Mean (SD)	31.3 (11.9)	29.7 (9.3)	0.95	0.34

^*1*^ Welch Two Sample t-test

**Table 10:** Comparison of hypertension service readiness scores in pharmacies with and without paramedics.

Characteristic	Pharmacy with paramedics, (N = 196)	Pharmacy without paramedics, (N = 109)	t-statistic	p-value^*1*^
**Hypertension Service Readiness**				
Guidelines and training, Mean (SD)	0.8 (6.2)	0.5 (4.8)	0.48	0.63
Essential equipment and supplies, Mean (SD)	64.4 (12.1)	62.2 (13.1)	1.43	0.16
Overall readiness score, Mean (SD)	32.6 (7.0)	31.3 (7.2)	1.45	0.15

^1^Welch Two Sample t-test

### Comparison of diabetes and hypertension service readiness among pharmacies of urban and non-urban pharmacies

Tables 11 and 12 show the comparison of diabetes and hypertension service readiness between rural/ peri-urban and urban pharmacies. Readiness scores for essential equipment for hypertension services across non-urban and urban settings were found to be statistically significant with rural/ peri-urban pharmacies having better score (ꭓ2 = 2.08, p = 0.04) suggesting association with the location of the pharmacies. Apart from that, there were no statistically significant differences across any readiness domains for both diabetes and hypertension services.

**Table 11:** Comparison of diabetes service readiness scores in urban and rural/peri-urban pharmacies.

Characteristic	Rural/ Peri-urban, (N = 55)	Urban, (N = 118)	t-statistic	p-value^*1*^
**Diabetes Service Readiness**				
Guidelines and training, Mean (SD)	0.9 (6.7)	1.3 (7.9)	−0.31	0.76
Essential equipment and supplies, Mean (SD)	71.3 (15.8)	69.5 (15.4)	0.70	0.49
Diagnosis, Mean (SD)	25.5 (30.2)	18.9 (22.4)	1.44	0.15
Overall readiness score, Mean (SD)	32.5 (12.3)	29.9 (10.3)	1.39	0.17

^1^Welch Two Sample t-test

**Table 12:** Comparison of hypertension service readiness scores in urban and rural/peri-urban pharmacies.

Characteristic	Rural/ Peri-urban, (N = 83)	Urban, (N = 222)	t-statistic	p-value^*1*^
**Hypertension Service Readiness**				
Guidelines and training, Mean (SD)	0.6 (5.5)	0.7 (5.8)	−0.10	0.92
Essential equipment and supplies, Mean (SD)	66.0 (12.4)	62.7 (12.5)	2.08	**0.04**
Overall readiness score, Mean (SD)	33.3 (7.1)	31.7 (7.1)	1.78	0.08

^1^Welch Two Sample t-test

## Discussion

This cross-sectional census survey aimed to assess the diabetes and hypertension services provided by private pharmacies and their readiness to deliver those services in PMC. To our knowledge, this is the first study to measure the readiness of the pharmacies and reveal whether pharmacies are capable to provide NCDs services.

Among all the private community pharmacies in PMC, a significant number of pharmacies are concentrated in the urban areas. The higher distribution of pharmacies in urban locality can be attributed to the substantial and growing urban population, which increased from 62.9% in 2011 to 66.1% in 2021 [42]. The greater density of population in the urban areas naturally generates the higher demand for the health care services. Additionally, urban areas offer more economic opportunities and higher income levels, making them more profitable and attractive locations for operating pharmacies. These factors collectively contribute to the higher concentration of pharmacies in urban areas.

About five percent of pharmacies were operated by non-pharmacist personnel in our study while the majority were managed by pharmacists and paramedic pharmacists. Although the number of pharmacies staffed by qualified pharmacists are increasing, some continue to be run by non-pharmacists. Previous studies have shown that, though the pharmacies have registered pharmacist, they are often operated by untrained staff or family members on an occasional basis [43]. This practice raises concerns, as it increases the risk of medication errors and inappropriate drug substitution. Pharmacists play a critical role in applying clinical judgement to identify potential drug interactions and contraindications, their absence can lead to adverse drug reactions or misuse of drugs. According to the National Good Pharmacy Practice, pharmacies should be operated either by pharmacists or assistant pharmacists [38]. However, our findings indicate that these standards are not being met, highlighting the need for stricter regulatory enforcement to ensure patient safety and maintain the professional standards.

The study found that the quality assurance activities were not conducted by all the pharmacies. This lack of consistent quality assurance has significant implications for patient safety and public health. One contributing factor may be the limited staffing and budgetary constraints commonly faced by pharmacies, which can affect the allocation of resources for quality assurance processes [44,45]. Additionally, the absence of standardized procedures for pharmacy quality assurance, along with insufficient regulatory oversight, further hinders the implementation of routine monitoring. To address these challenges, a standardized quality assurance protocol should be developed and implemented uniformly across all pharmacies.

Although it is mandatory for all pharmacies to be registered with the DDA under the Drug Act 2035 (Bikram Samvat) [46],our study found that 5.68% of pharmacies were operating without registration. This highlights a concerning gap in regulatory compliance that needs to be addressed. Enforcing mandatory registration is essential to safeguard patient safety and uphold the quality of the services.

In our study, only 10.8% of the surveyed pharmacies recorded data, indicating a very low level of documentation. This finding is consistent with a study conducted in Nigeria, which highlighted a lack of motivation among informal providers to maintain records [47]. One possible reason for this low uptake could be the absence of standardized recording system for use in private pharmacies. Although there are digitalized recording systems in hospital pharmacies in Nepal, it is limited to the recording of inventory management and procurement [46]. Previous study conducted in Nepal also noted that some community pharmacies, particularly in urban areas, have begun efforts to computerize their record, however, these electronic records are generally confined to inventory and storage purpose only [46]. The lack of reporting by pharmacies undermines the accuracy of the Health Management Information System (HMIS) data, as information from the pharmacies is not integrated into the current HMIS system. This issue prevents HMIS from accurately reflecting health service utilization across the country as the data of pharmacies are not included. Enhancing data recording practices such as the adoption of electronic systems would facilitate in the inclusion of pharmacy data in the broader scope of HMIS enabling better public health insights.

Our study revealed a gap in the linkage and collaboration between pharmacies and PMC. Strengthening this connection is essential, as collaboration with local authorities can help ensure pharmacies comply with regulatory standards and guidelines which can ultimately improve the quality of services. It is important that PMC takes initiative to offer support and coordination to facilitate stronger partnerships with private pharmacies.

The availability of hypertension services (87%) was greater than that of the diabetes services (49%) in our study. This disparity highlights the limited scope of services typically offered by the pharmacies in Nepal, which are primarily focused on dispensing prescription medications and providing instructions about intake of medicines [48]. Provisional diagnosis is the most common type of hypertension service offered by pharmacies and is provided based on the measurement of blood pressure only. In contrast, provisional diagnosis for diabetes requires additional resources such as blood glucose and urine test, which are not routinely available in most pharmacies. This likely explains the higher availability of hypertension services compared to diabetes services.

In our study the readiness score for the pharmacies regarding NCDs services are slightly lower in diabetes services compared to hypertension services. The overall mean score for the diabetes and hypertension service readiness was 30.7 and 32.1 respectively. Although the service readiness scores were relatively high for other domains, there were no guidelines available in any of the pharmacies, and few pharmacies had trained staff, both of which affect the overall readiness score. The mean readiness score for guidelines and training for hypertension was 0.7 which was lower than that of diabetes (1.2). This is consistent with previous research which has showed lower scores related to availability of guidelines for hypertension than diabetes, however we find lower scores in this domain than previous research. For instance, Adhikari et al (2023) reported scores of 12.3 and 14.9 for guidelines and training associated with hypertension and diabetes, respectively [34]. Their study, however, focused on health facilities more broadly, whereas our study specifically examined pharmacies, indicating that pharmacies have a lower level of readiness in this domain compared to other types of healthcare facilities. Additionally, the score for the staff training in this study was 4 for both the diabetes and hypertension services. This is a major concern, particularly given the complete absence of guidelines for managing these conditions. In contrast to this study, a study conducted in Bangladesh showed that the mean score for trained staff in private facilities were 5 for hypertension and 6 for diabetes services [49]. The lack of available guidelines in our study may reflect broader absence of national clinical guidelines for treatment of hypertension and poor implementation of NCDs policies into practice as mentioned in the previous study [34].

In contrast to findings from other studies where health facilities exceeded the standard readiness cutoff score [17,34,50], none of the pharmacies in our study met this benchmark. As a result, we report only the mean readiness scores for both the hypertension (32.1) and diabetes (30.7) services. Further analysis of National Health Facility Survey (NHFS) 2021 showed the median score for the readiness of local health facilities was 36.1 for both hypertension and DM services, which was similar to our study [34]. To our knowledge, this is the first study to assess the readiness and availability of NCDs services specifically within pharmacies in Nepal. Therefore, direct comparisons with other studies in the same context are not possible. However, the finding that pharmacies demonstrate a level of readiness similar to that of public health institutions suggest that they have the potential to play a significant role in NCD management particularly in areas with limited access to public health services. Strengthening the integration of pharmacies into the broader healthcare system, including the development of effective referral mechanisms between pharmacies and public health facilities, could ensure more comprehensive and continuous care for patients with hypertension and diabetes.

The findings of this study highlight several key areas that require further attention to enhance the quality and readiness of pharmacies in delivering diabetes and hypertension services. The government should consider implementing policies that prioritize improving public health facilities in rural areas, reducing the reliance on pharmacies for NCDs services. Strengthening public facilities in these areas can ensure equitable access to care without overburdening pharmacies. However, in urban areas, where the health system must increasingly rely on pharmacies to meet the needs of a growing population, efforts should focus on enhancing the capacity and integration of pharmacies into the healthcare system. This dual approach can address the challenges of both rural and urban settings, ensuring better access to NCD services for all communities. A major barrier identified is the lack of standardized guidelines and low levels of staff training for NCD services. This gap can be addressed through regular training and professional development initiatives such as the WHO PEN package. Local governments and professional associations could play a vital role in sponsoring these programs, thereby improving the readiness, accuracy and safety of pharmacy-based services for chronic conditions like hypertension and diabetes. The growing role of pharmacy in NCDs management and health screening is commendable, however, it presents challenges related to staff training, resource availability and funding. Expanding the scope of pharmacists practice to include preventive services such as routine health screenings and chronic disease monitoring may require policy reforms. Such reforms should address regulatory barriers and aim to foster collaboration and trust among healthcare stakeholders. Further, public health campaigns should focus on increasing awareness about the critical role of pharmacies in managing NCDs, helping people to access appropriate care in these settings.

This study has strengths such as the use of census data has provided comprehensive coverage of all the pharmacies of PMC, ensuring that the findings are representative and inclusive. Pharmacies are accessible healthcare points for the community people which offers essential health services and medications. Their growing influence indicates that they may eventually extend their services beyond dispensing drugs to include health screenings and chronic disease management. The results underline the necessity of formally establishing connections between pharmacies and other healthcare providers, offering crucial insights for policy makers. Policymakers may enhance healthcare delivery and accessibility by incorporating pharmacies into the larger healthcare system by acknowledging their function in healthcare system which can somewhat address the demand of the large urban population.

This study also has some limitations that should be considered such as the quality of services offered by pharmacies might vary across different settings and based on the providers. It is difficult to assess and ensure the service quality across all pharmacies for which there needs to be regular monitoring of pharmacies to ensure compliance with the established standards. The findings apply only to independent community pharmacies and cannot be generalized to hospital-based pharmacies because there may be different levels of readiness in services. If feasible, future research could expand to include hospital pharmacies for more comprehensive representation. Since the data were collected through interviews with the pharmacy staff, which may subject to reporting bias such as overreporting services to appear more competent which was minimized through observational validation wherever possible, but where it was not possible there might be the chance of reporting bias.

## Conclusion

Private pharmacies are increasingly involved in managing the hypertension and diabetes services in urban populations, often serving as a first contact point for the health system. Despite, delivering services related to diabetes and hypertension, the overall service readiness remains limited, particularly in the areas of standardized guidelines and staff training. In a context where paramedics of equivalent caliber in public health facilities are delivering the PEN package, its customized version can be adopted in pharmacies with similar cadres after capacitating them. Strengthening pharmacy competencies, strict regulation and more effective collaboration between pharmacies and public health bodies are necessary to overcome these issues, fill the current gap in service provision and ensure the consistent successful delivery of PEN program across settings. Integrating the pharmacies into the formal healthcare system could close key gaps in treating hypertension and diabetes among underserved and rural communities where hospital access is lacking. This integration would ensure more equitable and continuous access to essential NCDs services such as screening and lifestyle counselling for hypertension and diabetes. Furthermore, by recognizing and utilizing pharmacies as frontline healthcare providers, policymakers can leverage their broad reach and day to day interaction with communities to improve early detection, prevention, and long-term management of NCDs, ultimately strengthening the country’s preparedness to face growing burden of these diseases. The study findings have important implications for future research, such as longitudinal evaluations of the effectiveness of extended pharmacy services for NCDs management on long term patient outcomes (e.g., blood pressure control) and the potential for pharmacies to grow into more complete healthcare service providers. Monitoring and assessing the implementation of evidence-based interventions and evaluating their impact on service readiness, quality, and patient outcome will be key for future studies.

## Supporting information

S1 File Data. (XLSX)

S2 File Script. (TXT)
